# Prognostic value of preoperative inflammatory response biomarkers in patients with sarcomatoid renal cell carcinoma and the establishment of a nomogram

**DOI:** 10.1038/srep23846

**Published:** 2016-03-31

**Authors:** Liangyou Gu, Xin Ma, Hongzhao Li, Luyao Chen, Yongpeng Xie, Chaofei Zhao, Guoxiong Luo, Xu Zhang

**Affiliations:** 1 Department of Urology, Chinese PLA General Hospital, Beijing, China; 2 School of Medicine, Nankai University, Tianjin, China

## Abstract

To examine the prognostic role of inflammatory response biomarkers in sarcomatoid renal cell carcinoma (sRCC). From January 2004 to May 2015, 103 patients with sRCC were enrolled in this study. Preoperative neutrophil to lymphocyte ratio (NLR), derived neutrophil to lymphocyte ratio (dNLR), platelet to lymphocyte ratio (PLR) and lymphocyte to monocyte ratio (LMR) were analyzed. Besides well-established clinicopathological prognostic factors, we evaluated the prognostic value of this four markers using Kaplan-Meier method and Cox regression models. Additionally, a nomogram was established to predict the prognosis of sRCC patients. Elevated NLR, dNLR and PLR were significantly associated with worse overall survival (OS), nevertheless, elevated LMR showed an adverse effect on reduced OS. Multivariate analysis revealed that NLR (HR = 4.07, 95% CI = 1.50–11.00, *P* = 0.006) retained as independent factor. Incorporation of the NLR into a prognostic model including T stage, M stage, tumor necrosis and percentage of sarcomatoid generated a nomogram, which accurately predicted OS for sRCC patients. Preoperative NLR may serve as a potential prognostic biomarker in patients with sRCC and may help with clinical decisions about treatment intervention in clinical practice. The proposed nomogram can be used for the prediction of OS in patients with sRCC.

Sarcomatoid renal cell carcinoma (sRCC) is characterized by malignant spindle cells, similar to those present in sarcomas, within a background of epithelioid cells of renal cell carcinoma (RCC). First described by Farrow *et al.*^1^ in 1968, it was initially thought to represent a distinct entity of renal neoplasms. However, it can occur in all types of RCC and represent a common pathway of dedifferentiation of renal tumors^2^. Although about 5% of RCCs have sarcomatoid features, these cases account for a higher percentage of patients with advanced disease^3^. The patients with sRCC often have a worse prognosis compared with other patients with high-stage RCCs. By far, a few studies have attempted to risk-stratify patients with sarcomatoid RCC based on clinical and pathological features^4^,^5^,^6^,^7^,^8^; however, no commonly accepted prognostic model currently exists for this subset of patients. It is expected that a combination of specific RCC biomarkers into conventional clinicopathological parameters will allow better prediction of prognosis^9^.

Recently, emerging evidence indicates that inflammation plays a critical role in the initiation and progression of numerous cancers, including RCC^10^. In addition to local inflammatory symptoms, cancer patients frequently present with systemic inflammation responses, which is characterized by changes of peripheral blood cell amounts. Thus, several circulating blood cell-based prognostic biomarkers have been developed to predict patient outcome in various tumors, such as the neutrophil to lymphocyte ratio (NLR)^11^, derived neutrophil to lymphocyte ratio (dNLR)^12^, platelet to lymphocyte ratio (PLR)^13^ and lymphocyte to monocyte ratio (LMR)^14^. These markers are inexpensive to test and routinely measured in day-to-day clinical practice, and hence potentially provide readily available objective information to help doctors to estimate patient prognosis. However, only one or two inflammatory biomarkers have been evaluated for the prognosis of patients with RCC according to previous reports^14^,^15^. Moreover, the optimal cut-off values of the biomarkers from these studies were still discrepant.

To our knowledge, the prognostic value of inflammatory response biomarkers in sRCC has not been investigated. The current study aimed to investigate the prognostic role of these inflammatory biomarkers (NLR, dNLR, PLR, and LMR) in patients with sRCC, and was the first study attempting to establish a prognostic nomogram with improved predictive capacity for patients with sRCC based on these biomarkers and the clinicopathologic characteristics.

## Results

The clinicopathological characteristics of patients are shown in Table 1. After a median follow-up of 19.9 months (interquartile range, 10.8–35.1 months), 78 patients (75.7%) had died from all causes.

### The optimal cut-offs for NLR, dNLR, PLR and LMR

**Figure 1:**
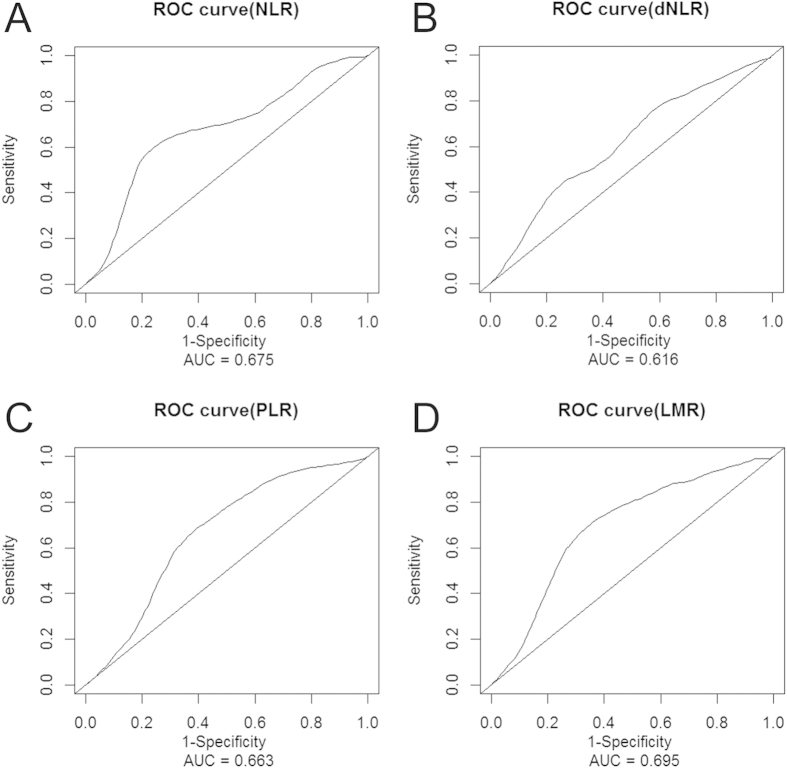
Optimal cut-off levels for NLR, dNLR, PLR and LMR were applied with ROC curves for overall survival (OS).

The ROC curves, using OS as the end-point for NLR, dNLR, PLR and LMR, were depicted in Fig. 1. The areas under curve (AUC) for NLR, dNLR, PLR and LMR were 0.675, 0.616, 0.663 and 0.695, respectively. The optimal cut-off levels were determined to be 4.10 for NLR, 2.57 for dNLR, 132 for PLR and 3.11 for LMR by ROC curves analysis. Patients were subsequently divided into two groups according to the optimal cut-off levels, with the high group ≥ the optimal cut-off levels and the low group that < the optimal cut-off levels.

### Associations of NLR, dNLR, PLR and LMR with OS

**Figure 2:**
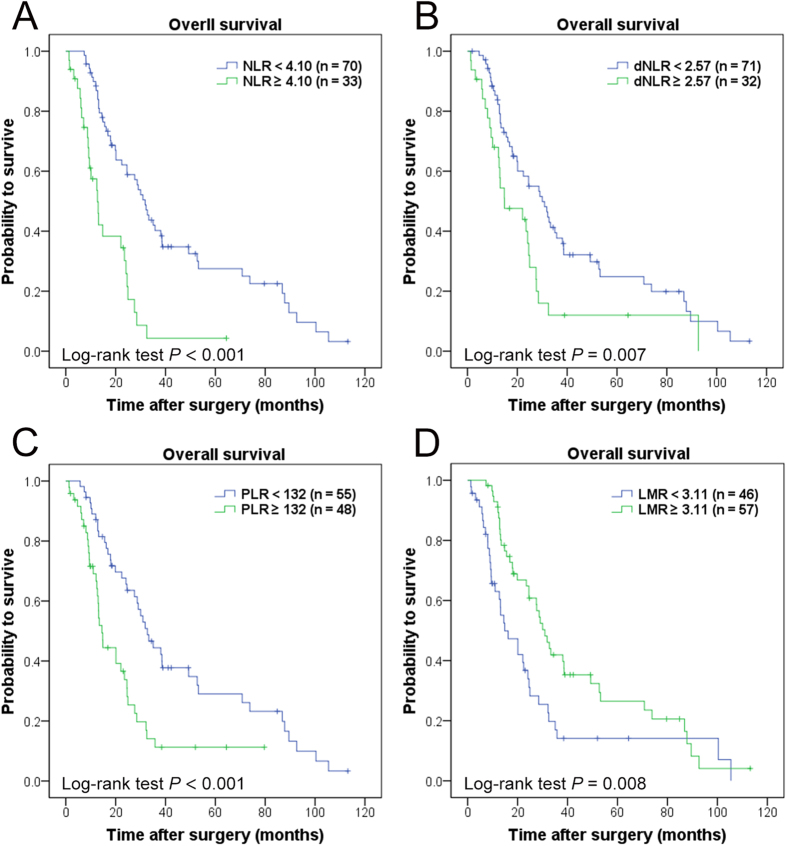
Kaplan-Meier curves for overall survival probability according to preoperative NLR, dNLR, PLR and LMR. Kaplan–Meier analysis for OS according to (**A**) preoperative NLR, (**B**) preoperative dNLR, (**C**) preoperative PLR, and (**D**) preoperative LMR.

To evaluate the associations, Kaplan-Meier survival analysis and log-rank tests were performed based on the postoperative survival time. Our results indicated that NLR (≥4.10) (*P* < 0.001), dNLR (≥2.57) (*P* = 0.007), PLR (≥132) (*P* < 0.001), LMR (<3.11) (*P* = 0.008) were significantly associated with decreased OS (Fig. 2A–D).

Inflammatory response biomarkers and clinicopathological parameters for the prediction of OS were further investigated by univariate analysis with Cox regression model. Results from the univariate analysis indicated that tumor size, T stage, N stage, M stage, histology, tumor necrosis, percentage of sarcomatoid differentiation, NLR, dNLR, PLR and LMR were prognostic factors of OS (Table 2). Then all of the 11 variables above were included in a multivariate Cox proportional hazards model to adjust the effects of covariates. In that model, we demonstrated that NLR was independent prognostic factor for OS (HR, 4.067; 95% CI, 1.504–10.995; *P* = 0.006), together with T stage (HR, 3.392; 95% CI, 1.885–6.103; *P* < 0.001), M stage (HR, 4.173; 95% CI, 2.256–7.719; *P* < 0.001), tumor necrosis (HR, 1.811; 95% CI, 1.066–3.077; *P* = 0.028) and percentage of sarcomatoid (HR, 2.244; 95% CI, 1.311–3,842; *P* = 0.003) (Table 2).

### Associations of NLR, dNLR, PLR and LMR with other clinicopathological variables

Potential relationships between NLR, dNLR, PLR and LMR and other clinicopathological factors were then explored (Table 3). Our results revealed that elevated NLR, dNLR and PLR were significantly correlated with tumor size >7 cm, high TNM stage (*P*_all_ < 0.05), separately. Moreover, both NLR and PLR were significantly higher in patients with a diagnosis of non-clear cell carcinoma compared with the clear cell group (*P* = 0.034 and 0.007, respectively). NLR and dNLR were both significantly higher in patients diagnosed with presence of tumor necrosis (*P* = 0.049 and 0.028, respectively). In addition, dNLR was significantly higher in female patients and patients with symptoms at presentation (*P* = 0.044 and 0.041, respectively), PLR was significantly higher in patients diagnosed with microvascular invasion (*P* = 0.013). By contrast, decreased LMR was significantly correlated with tumor size >7 cm, distant metastasis positivity, high TNM stage, presence of non- clear cell carcinoma and presence of tumor necrosis (*P*_all_ < 0.05), separately.

### Predictive nomogram for OS

**Figure 3:**
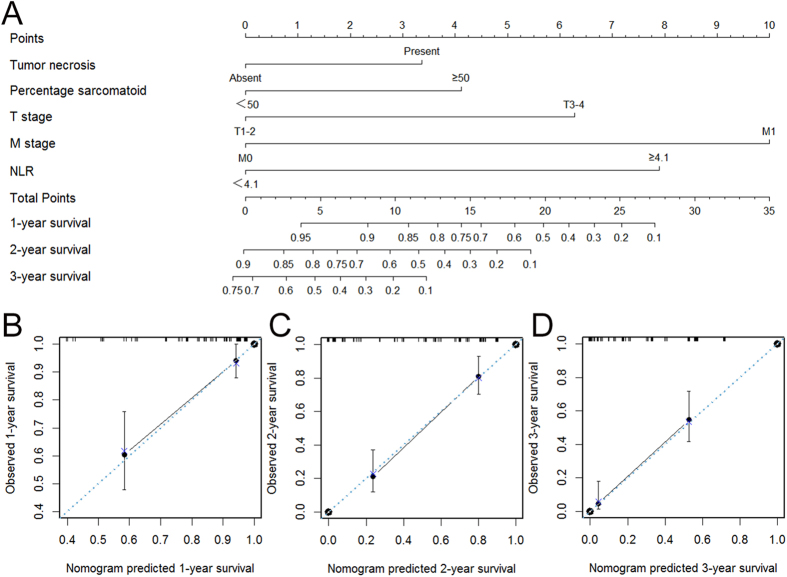
Nomogram for predicting 1-, 2- and 3-year OS of sRCC patients after nephrectomy. (**A**) Nomogram for predicting 1-, 2- and 3-year OS of sRCC patients after nephrectomy. Calibration plot of the nomogram for (**B**) 1-year, (**C**) 2-year and (**D**) 3-year survival. The blue dashed line represents the “ideal” line of a perfect match between predicted and observed survival. The black line indicates the performance of the proposed nomogram. Black dots are sub-cohorts of the data set; X is the bootstrapped corrected estimate of nomogram with 200 resamples. Vertical bars represent 95% confidence interval.

To predict the survival of sRCC patients after surgical resection, prognostic nomogram was depicted by Cox regression model analysis using all the significant independent indicators for OS consisting of T stage, M stage, tumor necrosis, percentage of sarcomatoid and NLR (Fig. 3A). The nomogram can predict the probability of death for sRCC patients within 1, 2 or 3 years after initial surgery. In this nomogram, a higher total point indicates a reduced OS. For internal validation, calibration plots of the nomogram predicting 1-, 2- and 3-year survival performed well with the ideal model (Fig. 3B–D). The C-index of the multivariate prognostic model based on T stage, M stage, tumor necrosis, percentage of sarcomatoid was 0.75 and improved to 0.78 when the NLR was incorporated.

## Discussion

Sarcomatoid differentiation has been shown as an adverse predictor of survival for patients with RCC in clinical practice. Multiple series have reported a median survival time of 4–19 months for patients with sRCC after diagnosis^4^,^5^,^6^,^7^. Recently, several studies have reported response of sRCC to anti-angiogenic therapy, although with limited efficacy. In particular, Golshayan *et al.*^16^ indicated among 43 patients who were treated with targeted therapy that over half of these patients achieved some degree of tumor shrinkage while on therapy. The median PFS and OS for these patients were reported to be 5.3 and 11.8 months, respectively. A more recent study by Molina *et al.*^17^ evaluated 63 patients with metastatic sRCC, of whom 29 were treated with sunitinib. A moderate improvement in PFS was noted compared with those who were treated with other therapies. Additionally, small clinical trials have shown that combined chemotherapy consisting of gemcitabine and doxorubicin has antitumor effect in patients with sRCC^18^,^19^. Although sarcomatoid differentiation is commonly recognized by clinicians to be associated with poor prognosis, a subset of patients has shown prolonged survival and favorable response to targeted therapy. Unfortunately, there is currently no commonly accepted prognostic model for sRCC. With this objective in mind, we attempt to evaluate the prognosis of patients with sRCC based on inflammatory response biomarkers and to establish a predictive nomogram to improve the predictive accuracy. To the best of our knowledge, this is the first report suggesting the prognostic value of systematic inflammation biomarkers in patients with sRCC.

As Rudolf Virchow initially made links between cancer and inflammation in the nineteenth century, contemporary studies have led to a general acceptance that inflammation has an important role in carcinogenesis^20^. Over the past decades, Virchow’s hypothesis was verified by emerging evidence showing the influence of inflammatory microenvironment on cancer. The inflammatory milieu facilitates tumor cells to evade host responses, contributing to angiogenesis, tumor growth, invasion, and metastasis. More understanding of the associations between inflammation and cancer contributes to the prevention and treatment of tumor. Recently, several biomarkers have been found to reflect the link between inflammation and RCC, such as platelet count^21^ and C-reactive protein^22^. Previous studies have shown that the systemic inflammatory biomarkers (NLR, dNLR, PLR, and LMR) can be also considered as potential prognostic factors for various types of carcinoma^12^,^13^,^14^,^23^.

The present study also showed that pre-operation NLR, dNLR, PLR and LMR in the peripheral blood of sRCC patients were significantly associated with tumor progression and poor prognosis after surgical resection. We demonstrated that increased NLR level before surgery was independent and adverse predictor of OS in multivariate analysis. Although dNLR, PLR and LMR were significantly associated with survival in univariate analysis, they were not retained as independent indicators in the multivariate model. The above results were supported by several mechanisms of inflammatory reaction to tumor.

Firstly, neutrophilia was triggered by cancer-related inflammatory factors, including granulocyte colony stimulating factor, tumor necrosis factor-alpha, interleukin-6, and myeloid growth factors^24^. Meantime, elevated circulating neutrophils have been particularly reported to contain and prompt secretion of the potent angiogenesis cytokine, namely, vascular endothelial growth factor (VEGF) and therefore accelerate tumor development^25^. Moreover, neutrophilia can secrete large amounts of reactive oxygen species (ROS), which cause cell DNA damage and genetic instability, inducing both carcinogenesis and promotion in tumor microenvironment^26^. Secondly, the decreased lymphocyte count and function play important roles in inflammatory reaction to tumor. Neutrophilia as an inflammatory response inhibits the immune system by suppressing the cytolytic activity of immune cells such as lymphocytes, activated T cells, and natural killer cells^27^. The importance of lymphocytes has been highlighted in several studies in which increasing infiltration of tumors with lymphocytes has been associated with better response to cytotoxic treatment and prognosis in cancer patients^28^,^29^. It has long been held that anti-tumor activity is mainly mediated by lymphocyte dependent cellular immune reactions. Lymphopenia is associated with diseases’ severity and immune escape of tumor cells from tumor-infiltrating lymphocytes. Additionally, an elevated NLR has been associated with an increase in the peritumoral infiltration of macrophages and an increase in interleukin (IL) 17^30^.

Some nomograms have been developed in various cancers, and nomograms have shown to be more accurate than the conventional staging systems for predicting prognosis in cancers^31^. The present study attempts to establish a predictive nomogram to predict the probability of postoperative patients who will die of sRCC within 1-year, 2-year and 3-year based on T stage, M stage, tumor necrosis, percentage of sarcomatoid and NLR. Calibration plots of the nomogram performed well in predicting OS with the ideal model, and the prediction was supported by C-index (0.78). These results supported that the nomogram could better predict overall survival in patients with sRCC post operation.

Several limitations of this study need to be acknowledged. Firstly, the study was a retrospective design, with a small population size of 103 patients, the prognostic significance of systematic inflammatory biomarkers in sRCC patients remains to be confirmed by prospective and clinical validation studies in the future. Also, further basic research studies will be performed to identify the detailed mechanism that how inflammatory cells and mediators are involved in the pathogenesis and progression of renal cell cancer. Secondly, the peripheral blood findings were not compared to the findings of peritumoral inflammation in the primary tumor tissue. Nevertheless, the peripheral blood results provide a novel horizon to understand the roles of inflammation in carcinogenesis and cancer progression. Thirdly, in the present study, the optimal cut-off levels of NLR, dNLR, PLR and LMR calculated by ROC curves based on OS. It was observed that the optimal cut-off values for these four markers were superior prognostic levels based on HR, which had the highest AUC and the best sensitivity and specificity. However, the optimal cutoff levels in this study was inconsistent with the results of previous studies, which may be due to the differences in assays measuring circulating blood cells, different population and different survival end-point. Fourthly, since CRP is not routinely tested in our clinical practice, we did not include CRP in our analyses. Further studies should evaluate the prognostic role of CRP in combination with other inflammatory biomarkers as well as clinicopathological parameters. Finally, there was some heterogeneity in the treatment used for patients after surgical resection, which led to different clinical prognosis. Therefore, further studies are needed to illuminate the relationship between inflammatory biomarkers and prognosis in patients with sRCC.

## Conclusions

In conclusion, preoperative NLR, dNLR, PLR and LMR are significantly associated with poor prognosis in sRCC patients. Moreover, NLR is an independent prognostic indicator for OS. The nomogram based on NLR and conventional clinicopathological variables could be used to accurately predict prognosis and offer optimal therapeutic strategy for patients with sRCC.

## Materials and Methods

### Patients and data collection

We retrospectively reviewed the medical records of patients with RCC who underwent radical or partial nephrectomy at our institution between January 2004 and May 2015. The inclusion criteria were as follows: 1) All patients with RCC and whose tumors had sarcomatoid features in the pathologic specimen; 2) No history of previous anti-cancer therapies and other malignancies; 3) No perioperative mortality; 4) No hematology disease, infection and hyperpyrexia; 5) Preoperative blood parameter data available; 5) Informed consents were obtained from eligible patients. At last, 103 patients were enrolled in the present study.

For each patient, the following clinical and pathologic information was gathered: age at surgery, gender, symptoms at presentation, nephrectomy pattern, tumor site and size, TNM stage^32^, histology, tumor necrosis, microvascular invasion and percentage of sarcomatoid differentiation. Histopathologic review on each of the tumor specimens was performed by a single pathologist to verify reported pathologic findings. The presentation mode was categorized as incidental or symptomatic. Tumors accompanied by fever or weight loss, pain, hematuria, abdominal mass were categorized as symptomatic tumors. Nephrectomy pattern was categorized as radical or partial. Tumor size was recorded as the longest diameter described in pathologic reports. The presence of nodal metastases was defined by pathologic examinations and the presence of distant metastases was defined according to radiographic findings. Histologic subtype was assigned based on recommendations of the International Union Against Cancer (UICC) and the American Joint Committee on Cancer (AJCC), and the Heidelberg classification system^33^. Tumor necrosis was defined as the presence of microscopic coagulative necrosis. Microvascular invasion refers to the presence of tumor within microscopic or veins with a muscular coat or the lymphatic system, or both. Percentage of sarcomatoid differentiation was estimated by reviewing all of the tumor sections microscopically, then classifying to <50% or ≥50%. The hematological parameters were obtained within 1 week before surgery. Preoperative NLR, dNLR, PLR and LMR were calculated from peripheral blood cell count. The definitions of them are described as follows: NLR = neutrophil to lymphocyte ratio; dNLR = neutrophil to (white cell count – neutrophil count) ratio; PLR = platelet count to lymphocyte ratio; and LMR = lymphocyte to monocyte ratio.

In our institute, patients were followed up every 3 to 6 months for the first 2 years after initial surgery, then annually. The typical items includes review of general health, physical examination, abdominal computed tomography or ultrasound, chest radiography, and laboratory exams. Follow-up was terminated in August 2015. This study was approved by Medical Ethics Committee of Chinese People’s Liberation Army General Hospital. The written informed consent was obtained from all subjects. The methods were carried out in accordance with the approved guidelines.

### Statistical analysis

Statistical analysis was performed with SPSS 20 (IBM Corporation, Armonk, NY, USA) and R 3.2.1 software (Institute for Statistics and Mathematics, Vienna, Austria). Overall survival (OS) was defined as the time from surgery to death from all causes. And patients were censored at end of follow-up, if death had not occurred. The optimal cut-off levels of NLR, dNLR, PLR, and LMR were determined by receiver operating curve (ROC) analysis (using the R software version 3.2.1). The end-point based on OS was applied. The Kolmogorov-Smirnov test revealed a non-normal distribution of NLR, dNLR, PLR, and LMR (each *P* < 0.05). Thus, they are shown as the median and IQR. Associations of NLR, dNLR, PLR, and LMR with other categorized clinicopathological prognostic variables were determined using nonparametric Wilcoxon rank-sum test or Kruskal-Wallis tests. Survival analyses on categorical variables were performed using the Kaplan-Meier method and significant differences between groups were identified using the log-rank test. The Cox proportional hazards regression model was applied to perform univariate and multivariate analyses, and those variables that achieved statistical significance in the univariate analysis were entered into the multivariable analysis. A nomogram for possible prognostic factors associated with OS was established by R software using ‘rms’ package. Calibration plots were generated to examine the performance characteristics of the predictive nomogram. The Harrell’s Concordance index (C-index) was used to evaluate the predictive accuracy^34^, which ranges from 0.5 (no predictive power) to 1 (perfect prediction). All statistical tests used in this study were two sided and *P*-values of <0.05 were considered significant.

## Additional Information

**How to cite this article**: Gu, L. *et al.* Prognostic value of preoperative inflammatory response biomarkers in patients with sarcomatoid renal cell carcinoma and the establishment of a nomogram. *Sci. Rep.* **6**, 23846; doi: 10.1038/srep23846 (2016).

## Figures and Tables

**Table 1:** Patient characteristics and pathological findings.

Characteristics	No. (%)
Age (years), Median (min-max)	56(16–79)
Gender	
Male	71(68.9)
Female	32(31.1)
Presentation	
Incidental	50(48.5)
Symptomatic	53(51.5)
Nephrectomy	
Radical	94(91.3)
Partial	9(8.7)
Tumor site	
Left	50(48.5)
Right	53(51.5)
Tumor size (cm)	
≤7	61(59.2)
>7	42(40.8)
T stage	
T1	44(42.7)
T2	26(25.2)
T3	29(28.2)
T4	4(3.9)
N stage	
N0	83(80.6)
N1	20(19.4)
M stage	
M0	76(73.8)
M1	27(26.2)
TNM stage	
I	32(31.1)
II	17(16.5)
III	27(26.2)
IV	27(26.2)
Histology	
Clear Cell	83(80.6)
Papillary	6(5.8)
Chromophobe	7(6.8)
Collecting duct	2(1.9)
Not otherwise specified (NOS)	5(4.9)
Tumor necrosis	
Absent	48(46.6)
Present	55(53.4)
Microvascular invasion	
Absent	82(79.6)
Present	21(20.4)
Percentage sarcomatoid (%)	
<50	63(61.2)
≥50	40(38.8)

No. = number of patients.

**Table 2:** Univariate and multivariate Cox proportional hazards regression analysis for OS.

Parameters	Univariate		Multivariate	
	HR (95% CI)	*P*-value	HR (95% CI)	*P*-value
Age (years)		0.125		
≤60	Reference			
>60	0.680(0.416–1.112)			
Gender		0.547		
Male	Reference			
Female	0.859(0.524–1.409)			
Presentation		0.419		
Incidental	Reference			
Symptomatic	0.832(0.533–1.300)			
Nephrectomy		0.953		
Radical	Reference			
Partial	1.028(0.412–2.569)			
Tumor site		0.072		
Left	Reference			
Right	1.510(0.964–2.367)			
Tumor size (cm)		0.045		0.805
≤7	Reference		Reference	
>7	1.614(1.012–2.574)		1.070(0.626–1.828)	
T stage		<0.001		<0.001
T1 + T2	Reference		Reference	
T3 + T4	3.088(1.812–5.264)		3.392(1.885–6.103)	
N stage		0.028		0.603
N0	Reference		Reference	
N1	1.820(1.068–3.103)		1.200(0.604–2.385)	
M stage		<0.001		<0.001
M0	Reference		Reference	
M1	4.282(2.562–7.158)		4.173(2.256–7.719)	
Histology		0.021		0.060
Clear Cell	Reference		Reference	
Non-clear Cell	1.875(1.099–3.201)		1.926(0.973–3.812)	
Tumor necrosis		0.012		0.028
Absent	Reference		Reference	
Present	1.821(1.144–2.900)		1.811(1.066–3.077)	
Microvascular invasion		0.084		
Absent	Reference			
Present	1.709(0.931–3.139)			
Percentage sarcomatoid (%)		0.035		0.003
<50	Reference		Reference	
≥50	1.615(1.033–2.525)		2.244(1.311–3.842)	
NLR		<0.001		0.006
<4.10	Reference		Reference	
≥4.10	3.259(1.962–5.412)		4.067(1.504–10.995)	
dNLR		0.008		0.283
<2.57	Reference		Reference	
≥2.57	1.926(1.189–3.120)		0.613(0.251–1.498)	
PLR		<0.001		0.627
<132	Reference		Reference	
≥132	2.386(1.482–3.840)		1.172(0.618–2.220)	
LMR		0.009		0.588
<3.11	Reference		Reference	
≥3.11	0.547(0.348–0.859)		0.850(0.472–1.531)	

HR = hazard ratio; CI = confidence interval; NLR = neutrophil count to lymphocyte count; dNLR = neutrophil count to (white cell count – neutrophil count); PLR = platelet count to lymphocyte count; LMR = lymphocyte to monocyte ratio.

**Table 3:** Associations of NLR, dNLR, PLR and LMR with other clinicopathological factors.

Factor	NLR, Median(IQR)	*P*	d-NLR, Median(IQR)	*P*	PLR, Median(IQR)	*P*	LMR, Median(IQR)	*P*
Total	3.07(2.20–4.78)		2.11(1.65–3.02)		122(85–183)		3.32(2.12–4.53)	
Age (years)		0.928		0.789		0.265		0.986
≤60	3.12(2.21–4.89)		2.11(1.64–3.27)		132(87–199)		3.34(2.10–4.52)	
>60	3.01(2.19–4.51)		2.13(1.70–2.69)		100(81–167)		3.27(2.61–4.72)	
Gender		0.480		0.044		0.875		0.853
Male	2.96(2.15–4.78)		2.03(1.59–2.57)		130(85–179)		3.38(2.00–4.53)	
Female	3.21(2.51–4.72)		2.33(1.84–3.34)		122(84–203)		3.23(2.44–4.68)	
Presentation		0.105		0.041		0.208		0.430
Incidental	2.94(1.96–4.39)		1.98(1.50–2.59)		121(72–180)		3.39(2.15–4.63)	
Symptomatic	3.17(2.51–4.96)		2.27(1.73–3.18)		138(94–192)		3.20(2.06–4.50)	
Tumor site		0.531		0.892		0.963		0.577
Left	2.99(2.46–4.80)		2.03(1.67–2.91)		126(84–177)		3.18(2.10–4.50)	
Right	3.16(1.98–4.64)		2.26(1.51–3.18)		122(79–197)		3.42(2.14–4.55)	
Tumor size (cm)		0.014		0.013		0.004		0.014
≤7	2.84(1.98–4.34)		2.03(1.52–2.53)		100(68–177)		3.54(2.66–4.86)	
>7	3.52(2.51–5.30)		2.29(1.80–3.54)		153(155–224)		2.83(2.00–3.65)	
T stage		0.283		0.174		0.302		0.930
T1 + T2	2.85(2.21–4.45)		1.98(1.65–2.73)		121(77–185)		3.26(2.11–4.73)	
T3 + T4	3.35(2.18–5.39)		2.48(1.67–3.27)		142(93–188)		3.39(2.41–4.12)	
N stage		0.701		0.471		0.214		0.739
N0	3.07(2.19–4.51)		2.12(1.63–2.70)		122(82–175)		3.35(2.11–4.72)	
N1	3.06(2.35–5.16)		2.13(1.70–3.29)		153(85–238)		3.21(2.23–4.21)	
M stage		0.245		0.254		0.062		0.044
M0	2.94(2.17–4.51)		1.98(1.64–2.70)		121(75–169)		3.43(2.24–4.76)	
M1	3.92(2.34–4.91)		2.18(1.76–3.27)		155(97–222)		2.74(2.04–3.54)	
TNM stage		0.022		0.044		0.001		0.006
I	2.64(1.91–3.38)		1.71(1.50–2.38)		87(57–137)		3.88(2.76–5.03)	
II	3.52(2.78–5.44)		2.26(1.77–3.26)		143(121–206)		2.49(1.81–3.42)	
III	3.17(2.20–5.66)		2.33(1.68–3.37)		122(85–192)		3.54(2.75–4.53)	
IV	3.92(2.34–4.91)		2.18(1.76–3.27)		155(97–222)		2.74(2.04–3.54)	
Histology		0.034		0.379		0.007		0.010
Clear Cell	2.86(2.15–4.74)		2.12(1.59–3.02)		121(73–167)		3.42(2.64–4.72)	
Non–clear Cell	3.65(2.80–5.41)		2.12(1.95–3.05)		179(106–247)		2.21(1.84–3.23)	
Tumor necrosis		0.049		0.028		0.058		0.025
Absent	2.85(2.03–4.11)		1.85(1.54–2.38)		115(69–178)		3.59(2.52–4.87)	
Present	3.43(2.51–4.98)		2.31(1.71–3.27)		139(95–216)		3.10(2.11–3.69)	
Microvascular invasion		0.167		0.297		0.013		0.089
Absent	2.91(2.18–4.57)		2.04(1.65–2.91)		120(73–180)		3.36(2.46–4.73)	
Present	3.51(2.61–6.12)		2.48(1.72–3.26)		163(121–226)		2.74(1.71–3.80)	
Percentage sarcomatoid (%)		0.771		0.552		0.710		0.561
<50	3.01(2.36–4.33)		2.11(1.66–2.69)		130(93–183)		3.35(2.00–4.07)	
≥50	3.25(2.05–5.40)		2.16(1.60–3.35)		122(70–190)		3.23(2.25–4.68)	

IQR = interquartile range; NLR = neutrophil count to lymphocyte count; dNLR = neutrophil count to (white cell count – neutrophil count); PLR = platelet count to lymphocyte count; LMR = lymphocyte to monocyte ratio.

## References

[b1] Farrow G. M., Harrison E. G. Jr. & Utz D. C. Sarcomas and sarcomatoid and mixed malignant tumors of the kidney in adults. 3. Cancer 22, 556–563 (1968). 4299778 10.1002/1097-0142(196809)22:3<556::aid-cncr2820220310>3.0.co;2-n

[b2] Delahunt B. Sarcomatoid renal carcinoma: the final common dedifferentiation pathway of renal epithelial malignancies. Pathology 31, 185–190 (1999). 10503259 10.1080/003130299104945

[b3] Shuch B. *et al.* Quality of pathological reporting for renal cell cancer: implications for systemic therapy, prognostication and surveillance. BJU international 108, 343–348, 10.1111/j.1464-410X.2010.09871.x (2011). 21087450

[b4] de Peralta-Venturina M. *et al.* Sarcomatoid differentiation in renal cell carcinoma: a study of 101 cases. The American journal of surgical pathology 25, 275–284 (2001). 11224597 10.1097/00000478-200103000-00001

[b5] Mian B. M. *et al.* Prognostic factors and survival of patients with sarcomatoid renal cell carcinoma. The Journal of urology 167, 65–70 (2002). 11743277

[b6] Cheville J. C. *et al.* Sarcomatoid renal cell carcinoma: an examination of underlying histologic subtype and an analysis of associations with patient outcome. The American journal of surgical pathology 28, 435–441 (2004). 15087662 10.1097/00000478-200404000-00002

[b7] Shuch B. *et al.* Impact of pathological tumour characteristics in patients with sarcomatoid renal cell carcinoma. BJU international 109, 1600–1606, 10.1111/j.1464-410X.2011.10785.x (2012). 22221668 PMC4676716

[b8] Zhang B. Y. *et al.* A novel prognostic model for patients with sarcomatoid renal cell carcinoma. BJU international 115, 405–411, 10.1111/bju.12781 (2015). 24730416

[b9] Shariat S. F. & Xylinas E. Biomarkers in personalised treatment of renal-cell carcinoma. The lancet oncology 13, 751–752, 10.1016/s1470-2045(12)70292-9 (2012). 22759479

[b10] Mantovani A., Allavena P., Sica A. & Balkwill F. Cancer-related inflammation. Nature 454, 436–444, 10.1038/nature07205 (2008). 18650914

[b11] Pichler M. *et al.* Validation of the pre-treatment neutrophil-lymphocyte ratio as a prognostic factor in a large European cohort of renal cell carcinoma patients. British journal of cancer 108, 901–907, 10.1038/bjc.2013.28 (2013). 23385728 PMC3590665

[b12] Dalpiaz O. *et al.* Validation of the pretreatment derived neutrophil-lymphocyte ratio as a prognostic factor in a European cohort of patients with upper tract urothelial carcinoma. British journal of cancer 110, 2531–2536, 10.1038/bjc.2014.180 (2014). 24691424 PMC4021523

[b13] Templeton A. J. *et al.* Prognostic role of platelet to lymphocyte ratio in solid tumors: a systematic review and meta-analysis. Cancer epidemiology, biomarkers & prevention : a publication of the American Association for Cancer Research, cosponsored by the American Society of Preventive Oncology 23, 1204–1212, 10.1158/1055-9965.epi-14-0146 (2014). 24793958

[b14] Hutterer G. C. *et al.* Low preoperative lymphocyte-monocyte ratio (LMR) represents a potentially poor prognostic factor in nonmetastatic clear cell renal cell carcinoma. Urologic oncology 32, 1041–1048, 10.1016/j.urolonc.2014.04.001 (2014). 25027686

[b15] de Martino M. *et al.* Prognostic impact of preoperative neutrophil-to-lymphocyte ratio in localized nonclear cell renal cell carcinoma. The Journal of urology 190, 1999–2004, 10.1016/j.juro.2013.06.082 (2013). 23831313

[b16] Golshayan A. R. *et al.* Metastatic sarcomatoid renal cell carcinoma treated with vascular endothelial growth factor-targeted therapy. Journal of clinical oncology : official journal of the American Society of Clinical Oncology 27, 235–241, 10.1200/jco.2008.18.0000 (2009). 19064974

[b17] Molina A. M. *et al.* Sarcomatoid-variant renal cell carcinoma: treatment outcome and survival in advanced disease. American journal of clinical oncology 34, 454–459, 10.1097/COC.0b013e3181f47aa4 (2011). 21127411 PMC3661202

[b18] Roubaud G. *et al.* Combination of gemcitabine and doxorubicin in rapidly progressive metastatic renal cell carcinoma and/or sarcomatoid renal cell carcinoma. Oncology 80, 214–218, 10.1159/000329078 (2011). 21720184

[b19] Haas N. B. *et al.* A phase II trial of doxorubicin and gemcitabine in renal cell carcinoma with sarcomatoid features: ECOG 8802. Medical oncology (Northwood, London, England) 29, 761–767, 10.1007/s12032-011-9829-8 (2012). PMC3566570 21298497

[b20] Balkwill F. & Mantovani A. Inflammation and cancer: back to Virchow? Lancet 357, 539–545, 10.1016/s0140-6736(00)04046-0 (2001). 11229684

[b21] Gu L. *et al.* The association of platelet count with clinicopathological significance and prognosis in renal cell carcinoma: a systematic review and meta-analysis. PloS one 10, e0125538, 10.1371/journal.pone.0125538 (2015). 25955026 PMC4425534

[b22] Hu Q. *et al.* The prognostic value of C-reactive protein in renal cell carcinoma: a systematic review and meta-analysis. Urologic oncology 32, 50 e51–58, 10.1016/j.urolonc.2013.07.016 (2014). 24239465

[b23] Templeton A. J. *et al.* Prognostic role of neutrophil-to-lymphocyte ratio in solid tumors: a systematic review and meta-analysis. Journal of the National Cancer Institute 106, dju124, 10.1093/jnci/dju124 (2014). 24875653

[b24] Lord B. I. *et al.* The kinetics of human granulopoiesis following treatment with granulocyte colony-stimulating factor *in vivo*. Proceedings of the National Academy of Sciences of the United States of America 86, 9499–9503 (1989). 2480603 10.1073/pnas.86.23.9499 PMC298524

[b25] Jablonska J., Leschner S., Westphal K., Lienenklaus S. & Weiss S. Neutrophils responsive to endogenous IFN-beta regulate tumor angiogenesis and growth in a mouse tumor model. The Journal of clinical investigation 120, 1151–1164, 10.1172/jci37223 (2010). 20237412 PMC2846036

[b26] Weitzman S. A. & Gordon L. I. Inflammation and cancer: role of phagocyte-generated oxidants in carcinogenesis. Blood 76, 655–663 (1990). 2200535

[b27] Petrie H. T., Klassen L. W. & Kay H. D. Inhibition of human cytotoxic T lymphocyte activity *in vitro* by autologous peripheral blood granulocytes. Journal of immunology (Baltimore, Md. : 1950) 134, 230–234 (1985). 3871101

[b28] Denkert C. *et al.* Tumor-associated lymphocytes as an independent predictor of response to neoadjuvant chemotherapy in breast cancer. Journal of clinical oncology : official journal of the American Society of Clinical Oncology 28, 105–113, 10.1200/jco.2009.23.7370 (2010). 19917869

[b29] Gooden M. J., de Bock G. H., Leffers N., Daemen T. & Nijman H. W. The prognostic influence of tumour-infiltrating lymphocytes in cancer: a systematic review with meta-analysis. British journal of cancer 105, 93–103, 10.1038/bjc.2011.189 (2011). 21629244 PMC3137407

[b30] Motomura T. *et al.* Neutrophil-lymphocyte ratio reflects hepatocellular carcinoma recurrence after liver transplantation via inflammatory microenvironment. Journal of hepatology 58, 58–64, 10.1016/j.jhep.2012.08.017 (2013). 22925812

[b31] Balachandran V. P., Gonen M., Smith J. J. & DeMatteo R. P. Nomograms in oncology: more than meets the eye. The lancet oncology 16, e173–180, 10.1016/s1470-2045(14)71116-7 (2015). 25846097 PMC4465353

[b32] Edge S. B. & Compton C. C. The American Joint Committee on Cancer: the 7th edition of the AJCC cancer staging manual and the future of TNM. Annals of surgical oncology 17, 1471–1474, 10.1245/s10434-010-0985-4 (2010). 20180029

[b33] Kovacs G. *et al.* The Heidelberg classification of renal cell tumours. The Journal of pathology 183, 131–133, 10.1002/(sici)1096-9896(199710)183:2<131::aid-path931>3.0.co;2-g(1997 ). 9390023

[b34] Harrell F. E. Jr., Lee K. L. & Mark D. B. Multivariable prognostic models: issues in developing models, evaluating assumptions and adequacy, and measuring and reducing errors. Statistics in medicine 15, 361–387, 10.1002/(sici)1097-0258(19960229)15:4<361::aid-sim168>3.0.co;2–4(1996 ). 8668867

